# Supplementary figures and images for: Mutational analysis of Phanerochaete chrysosporium´s purine transporter
Source: PLoS One. 2024 Oct 31;19(10):e0313174. doi: 10.1371/journal.pone.0313174 (PMC11527162; doi:10.1371/journal.pone.0313174)

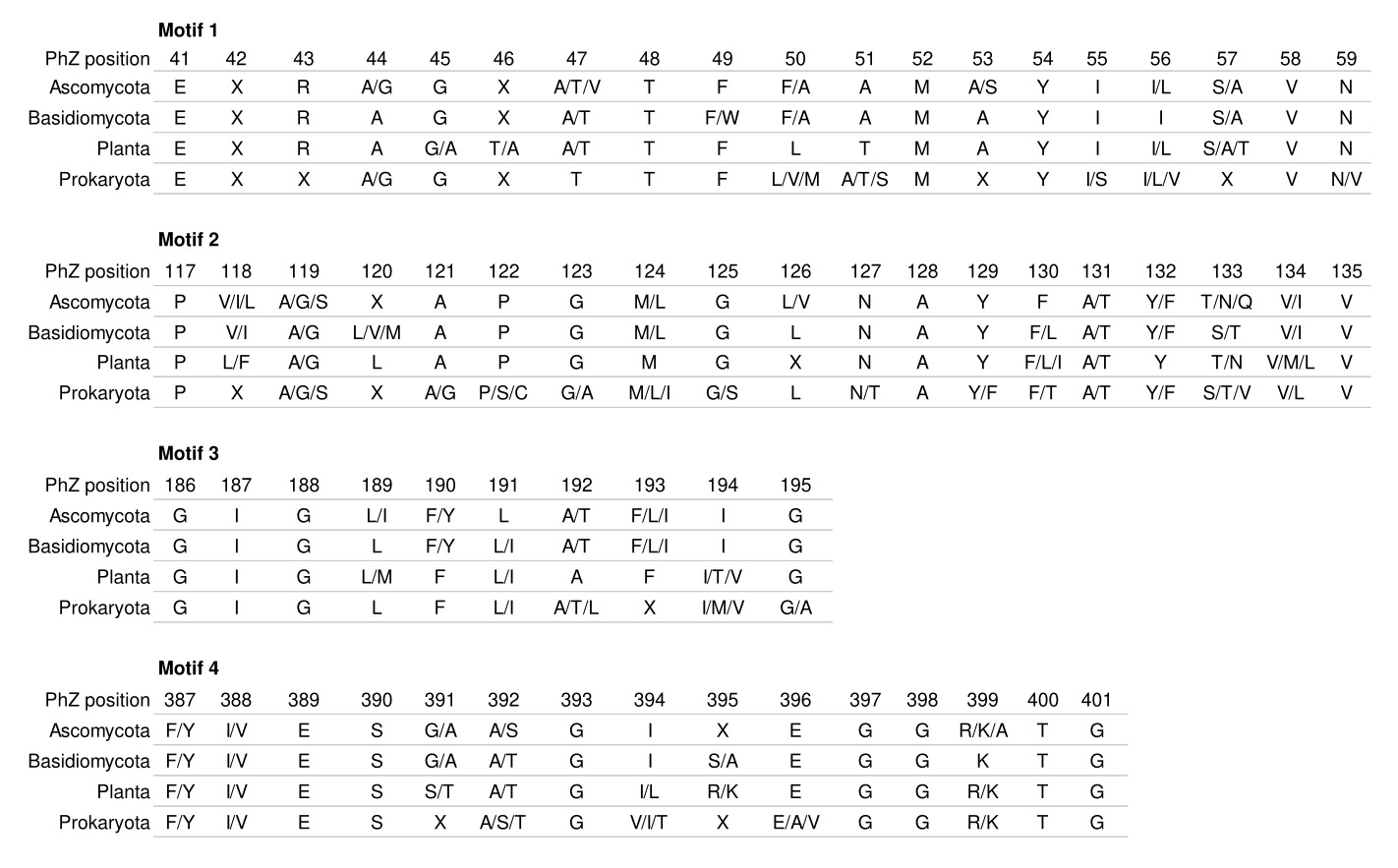

Supplement: S1 Fig — The analysis was based on the alignment obtained through ClustalW of AzgA-like proteins with known functions and 144 hypothetical ones (S1 Appendix). The numbering of the marked amino acids corresponds to the PhZ sequence. X represents any amino acid. (TIF) [file pone.0313174.s001.tif]

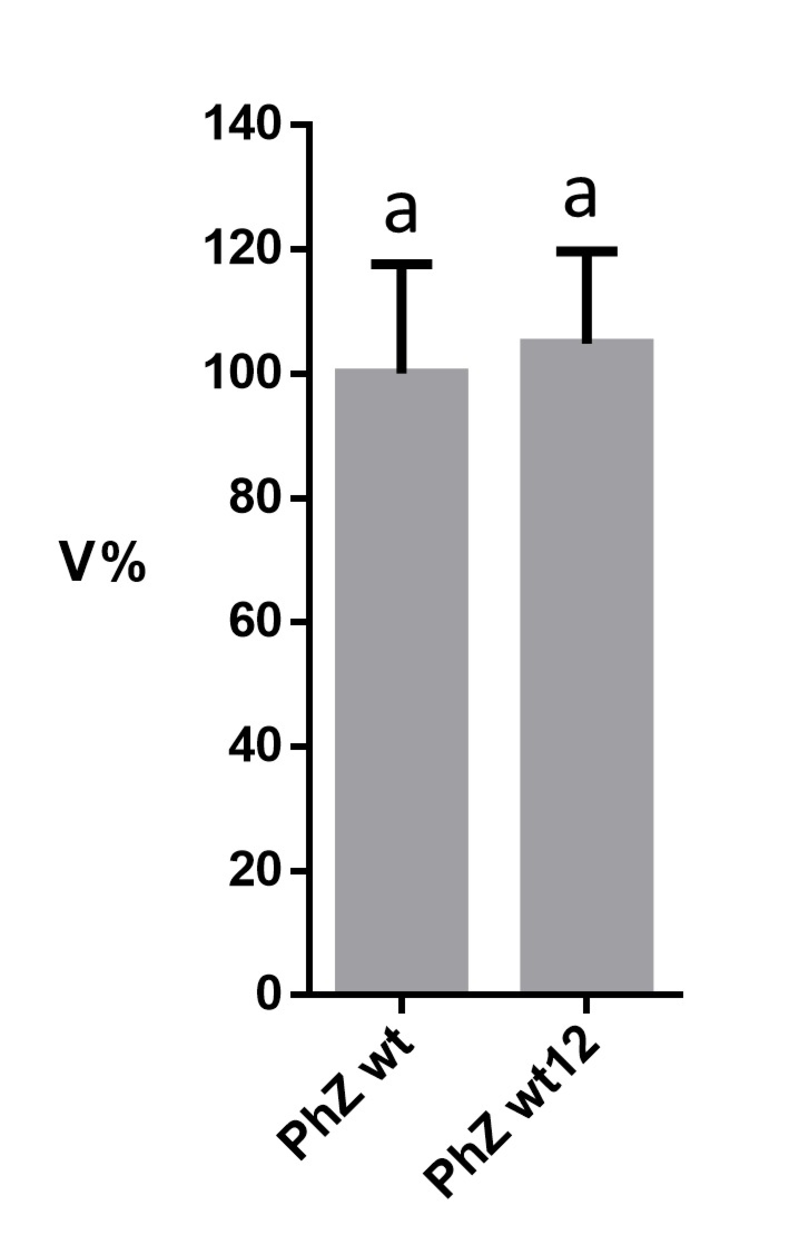

Supplement: S2 Fig — Strains PhZwt and PhZwt12 expressing the GFP- and non-GFP transporter, respectively, are included. 100% is considered the transport rate of the PhZwt strain. Results are the average of two independent experiments each measured in triplicate. Identical letters (a) indicate no significant differences were found according to ANOVA and Tukey’s test (p = 0.616). (TIF) [file pone.0313174.s002.tif]

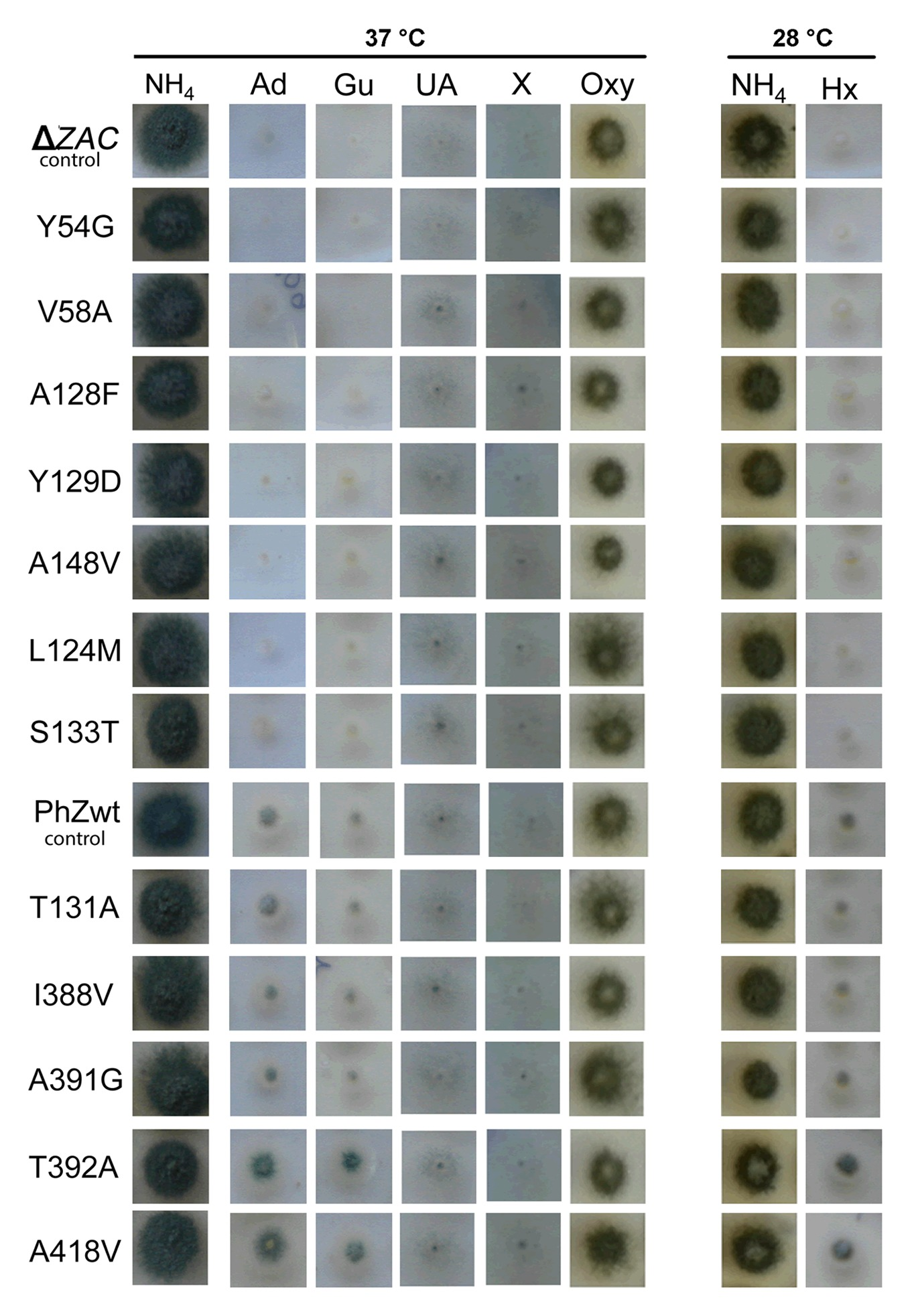

Supplement: S3 Fig — This figure complements Fig 3. Left panel: growth in the presence of ammonium L(+) tartrate (NH4), adenine (Ad), guanine (Gu), uric acid (UA), xanthine (X), and oxypurinol + 10 mM sodium nitrate (Oxy) at 37°C for 48 hours. Right panel: growth on ammonium (NH4) and hypoxanthine (Hx) at 28°C for 4 days. Control strains: ΔZAC and PhZwt. (TIF) [file pone.0313174.s003.tif]

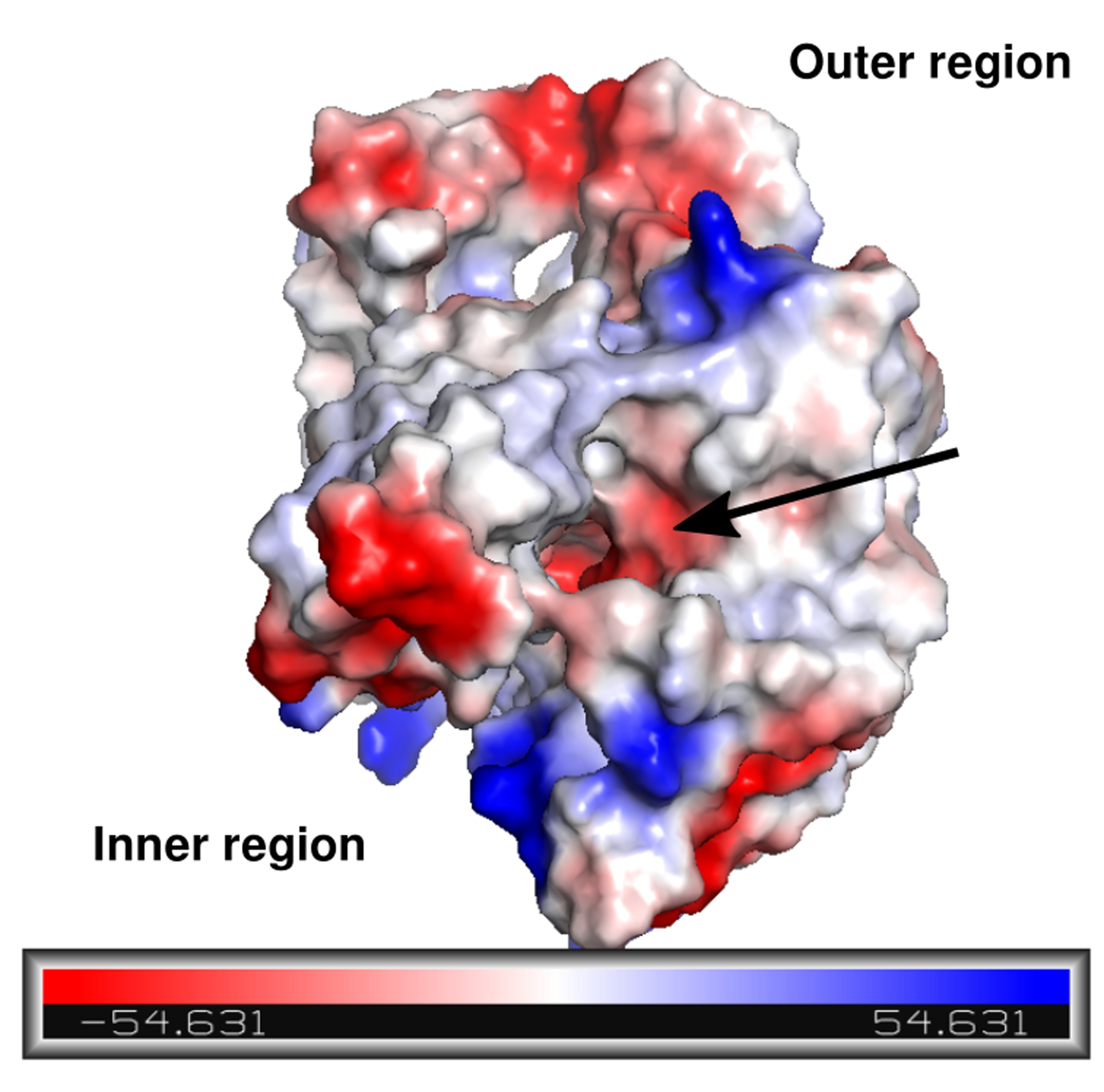

Supplement: S4 Fig — The charge is indicated by the colour bar in the figure. A highly negatively charged red area, which would be part of the protein’s pore, in the inner area between TMS8, TMS5, and internal helix 1, is indicated with an arrow. (TIF) [file pone.0313174.s004.tif]

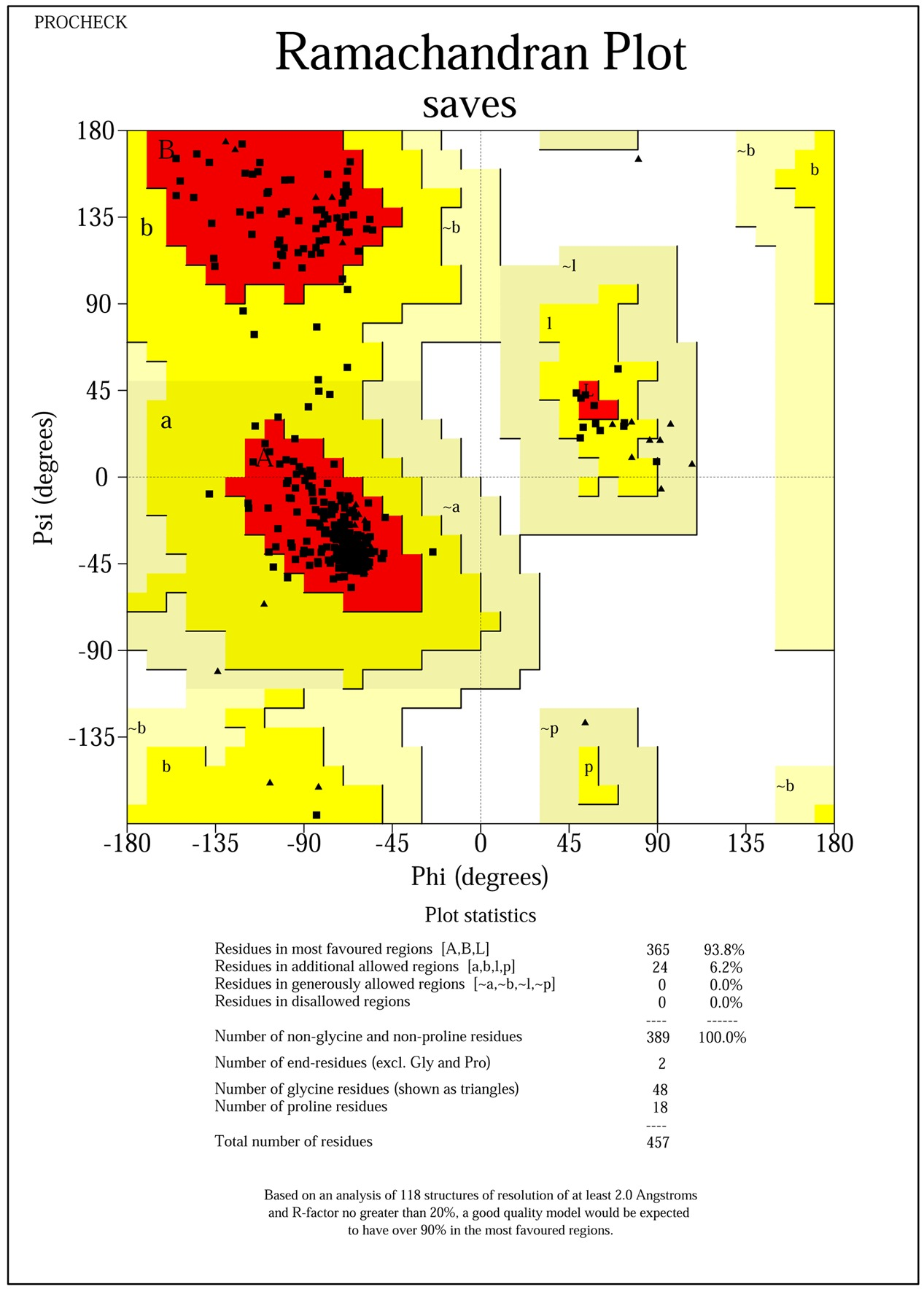

Supplement: S5 Fig — The plot shows the distribution of φ (phi) and ψ (psi) angles for the amino acid residues in PhZ wt. Analysis performed using PROCHECK. (TIF) [file pone.0313174.s005.tif]

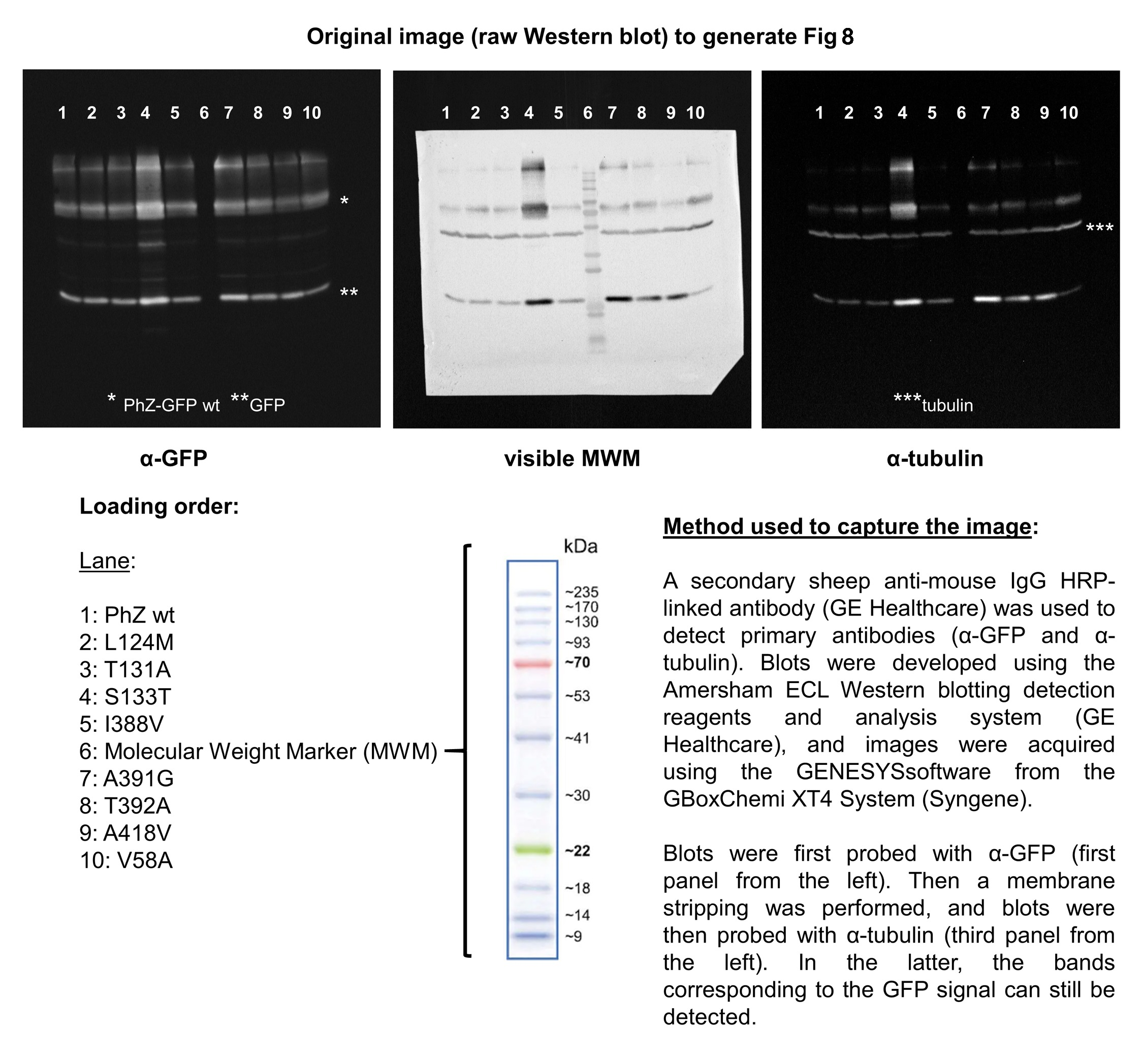

Supplement: S1 Raw image — (TIF) [file pone.0313174.s009.tif]
