# Supplementary material for: Mutational analysis of Phanerochaete chrysosporium´s purine transporter
Source: PLoS One. 2024 Oct 31;19(10):e0313174. doi: 10.1371/journal.pone.0313174 (PMC11527162; doi:10.1371/journal.pone.0313174)
Supplement: S1 Table — (DOCX) [file pone.0313174.s006.docx]

| Name / Collection number | Genotype (all strains carry the *veA1* mutation ) |
| --- | --- |
| ANwt / MVD001 | *pabaA1* |
| *ΔZAC* / MV060 [17] | *ΔazgA ΔuapA ΔuapCΔ::pyrG^AF^ ΔnkuA pabaA1 riboB2 pyroA4* |
| PhZwt / AN40Z42 [17] | *ΔazgA::phZ::gfp::riboB ΔuapAΔuapC::pyrG^AF^ ΔnkuA::argB pabaA1 riboB2 pyroA4* |
| PhZwt12 / AN40Z12 [17] | *ΔazgA::phZ::riboB ΔuapAΔuapC::pyrG^AF^ ΔnkuA::argB pabaA1 riboB2 pyroA4* |
| A*zgA* / AN35AZ121 [17] | *azgA::gfp::riboB ΔuapAΔuapC::pyrG^AF^ ΔnkuA::argB pabaA1 riboB2 pyroA4* |
| phZ_Y54G* | *ΔazgA::phZY54G::gfp::riboBΔuapAΔuapC::pyrG^AF^ ΔnkuA::argB pabaA1 riboB2 pyroA4* |
| phZ_V58A* | *ΔazgA::phZV58A::gfp::riboBΔuapAΔuapC::pyrG^AF^ ΔnkuA::argB pabaA1 riboB2 pyroA4* |
| phZ_A128F* | *ΔazgA::phZA128F::gfp::riboBΔuapAΔuapC::pyrG^AF^ ΔnkuA::argB pabaA1 riboB2 pyroA4* |
| phZ_Y129D* | *ΔazgA::phZY129D::gfp::riboBΔuapAΔuapC::pyrG^AF^ ΔnkuA::argB pabaA1 riboB2 pyroA4* |
| phZ_A148V* | *ΔazgA::phZA148V::gfp::riboBΔuapAΔuapC::pyrG^AF^ ΔnkuA::argB pabaA1 riboB2 pyroA4* |
| phZ_A418V* | *ΔazgA::phZ A418V::gfp::riboBΔuapAΔuapC::pyrG^AF^ ΔnkuA::argB pabaA1 riboB2 pyroA4* |
| phZ_T429P* | *ΔazgA::phZT429P::gfp::riboBΔuapAΔuapC::pyrG^AF^ ΔnkuA::argB pabaA1 riboB2 pyroA4* |
| phZ_L124M* | *ΔazgA::phZL124M::gfp::riboBΔuapAΔuapC::pyrG^AF^ ΔnkuA::argB pabaA1 riboB2 pyroA4* |
| phZ_T131A* | *ΔazgA::phZT131A::gfp::riboBΔuapAΔuapC::pyrG^AF^ ΔnkuA::argB pabaA1 riboB2 pyroA4* |
| phZ_S133T* | *ΔazgA::phZS133T::gfp::riboBΔuapAΔuapC::pyrG^AF^ ΔnkuA::argB pabaA1 riboB2 pyroA4* |
| phZ_I388V* | *ΔazgA::phZI388V::gfp::riboBΔuapAΔuapC::pyrG^AF^ ΔnkuA::argB pabaA1 riboB2 pyroA4* |
| phZ_A391G* | *ΔazgA::phZA391G::gfp::riboBΔuapAΔuapC::pyrG^AF^ ΔnkuA::argB pabaA1 riboB2 pyroA4* |
| phZ_T392A* | *ΔazgA::phZT392A::gfp::riboBΔuapAΔuapC::pyrG^AF^ ΔnkuA::argB pabaA1 riboB2 pyroA4* |

**S1 Table. *Aspergillus nidulans* strains used in this study**

*Obtained in this study
