# Supplementary material for: Mutational analysis of Phanerochaete chrysosporium´s purine transporter
Source: PLoS One. 2024 Oct 31;19(10):e0313174. doi: 10.1371/journal.pone.0313174 (PMC11527162; doi:10.1371/journal.pone.0313174)
Supplement: S2 Table — (DOCX) [file pone.0313174.s007.docx]

| Primer | sequence | Mutation |
| --- | --- | --- |
| mphZ_Y54G_F1 | TGGGCCGCCATGGCGggCATCATCTCGGTG | Y54G |
| mphZ_Y54G_R1 | CACCGAGATGATGccCGCCATGGCGGCCCA |  |
| mphZ_V58A_F | CGTACATCATCTCGGcGAACGCCTCCATCATCTCG | V58A |
| mphZ_V58A_R | CGAGATGATGGAGGCGTTCgCCGAGATGATGTACG |  |
| mphZ_A128F_F2 | GGACTCGGGTTGAACttcTACTTCACATACTCGGTTGTTGGT | A128F |
| mphZ_A128F_R2 | ACCAACAACCGAGTATGTGAAGTAGAAGTTCAACCCGAGTCC |  |
| mphZ_Y129D_F3 | GACTCGGGTTGAACGCAgACTTCACATACTCGGTTG | Y129D |
| mphZ_Y129D_R3 | CAACCGAGTATGTGAAGTcTGCGTTCAACCCGAGTC |  |
| mphZ_A148V_F | CATCACGTACCGCGAGGCtCTCGCTGCGGTCTTCCTTG | A148V |
| mphZ_A148V_R | CAAGGAAGACCGCAGCGaGAGCCTCGCGGTACGTGATG |  |
| mphZ_A418V_F | CTTCATCAGCGTGTTCTTCGtCCCGATCTTTGCGAGCAT | A418V |
| mphZ_A418V_R | ATGCTCGCAAAGATCGGGaCGAAGAACACGCTGATGAAG |  |
| mphZ_T429P_F | CATCCCCTCCTGGGCTcCAGGCGGTGCCCTCG | T429P |
| mphZ_T429P_R | CGAGGGCACCGCCTGgAGCCCAGGAGGGGATG |  |
| mphZ _L124M_F1 | ATGGCGCCCGGAaTgGGGTTGAACGCATAC | L124M |
| mphZ _L124M_R1 | TATGCGTTCAACCCcAtTCCGGGCGCCATG |  |
| mphZ _T131A_F2 | AACGCATACTTCgCcTACTCGGTTGTTGGTTTCCAC | T131A |
| mphZ _T131A_R2 | TGGAAACCAACAACCGAGTAcGcGAAGTATGCGTTC |  |
| mphZ _S133T_F3 | GTTGAACGCATACTTCACATACaCtGTTGTTGGTTTCCAC | S133T |
| mphZ _S133T_R3 | GTGGAAACCAACAACaGtGTATGTGAAGTATGCGTTCAAC |  |
| mphZ _I388V_F5 | TGTCACCGCGTTCgTCGAGTCCGCG | I388V |
| mphZ _I388V_R5 | GGACTCGAcGAACGCGGTGACAGGG |  |
| mphZ _A391G_F6 | GTTCATCGAGTCCGgtACCGGTATCTCCGAG | A391G |
| mphZ _A391G_R6 | TCGGAGATACCGGTACCGGACTCGATGAACG |  |
| mphZ _T392A_F7 | ATCGAGTCCGCGgCCGGTATCTCCG | T392A |
| mphZ _T392A_R7 | GGAGATACCGGcCGCGGACTCGATG |  |

**S2 Table. Primers used in this study**
