# Supplementary material for: Mutational analysis of Phanerochaete chrysosporium´s purine transporter
Source: PLoS One. 2024 Oct 31;19(10):e0313174. doi: 10.1371/journal.pone.0313174 (PMC11527162; doi:10.1371/journal.pone.0313174)
Supplement: S1 Appendix — (DOCX) [file pone.0313174.s008.docx]

**S1 Appendix. Accession number of the sequences used in this study.**

**Proteins of known function**

AOC89074.1 (*Phanerochaete chrysosporium* PhZ), XP_681803, CAA50681.2 (*Aspergillus nidulans* AzgA, UapA)*,* AFUA_5G09750 *(Aspergillus fumigatus* AfAzgA)*,* NP_566384.1, OAO91377.1 (*Arabidopsis thaliana* Atzg1, Atzg2), P31466.2, NJV00898.1, AGX33403.1, EFN5098773.1 *(Escherichia coli* PurP, YicO, YjcD, YgfQ)*,* NP_047008.1, NP_047009 (*Borellia burgdorferi* BBB22, BBB23).

**Hypothetical proteins**

Plants

BAJ90773.1 (*Hordeum vulgare*), EAY97568.1 (*Oryza sativa*), XP_004502854.1 (*Cicerarietinum* sp), XP_002979897.1 (*Selaginella moellendorffii*), CAN76560.1 (*Vitis vinifera*), XP_006435876.1 (*Citrus clementina*), XP_006361315.1 (*Solanum tuberosum*), EPS66133.1 (*Genlisea aurea*), AAM63152.1 (*Arabidopsis thaliana*), XP_001769041.1 (*Physcomitrella patens*), XP_006407485.1 (*Eutrema salsugineum*), XP_007137610.1 (*Phaseolus vulgaris*), XP_006581731.1 (*Glycine max*), XP_002533721.1 (*Ricinus communis*), EXB96512.1 (*Morus notabilis*), XP_004307720.1 (*Fragaria vesca*), EYU20110.1 (*Mimulus guttatus*), XP_002323060.1 (*Populus trichocarpa*), XP_004138552.1 (*Cucumis sativus*), XP_004236321.1 (*Solanum lycopersicum*), XP_006842760.1 (*Amborella trichopoda*), EMS47128.1 (*Triticum urartu*), NP_001141828.1 (*Zea mays*), XP_004962437.1 (*Setaria italica*), XP_007033567.1 (*Theobroma cacao*), EMT29089.1 (*Aegilops tauschii*), XP_004956378.1 (*Setaria italica*), XP_002450589.1 (*Sorghum bicolor*), XP_003577601.1 (*Brachypodium distachyon*), XP_007209883.1 (*Prunus persica*), XP_006477555.1 (*Citrus sinensis*), XP_006663393.1 (*Oryza Brachyantha*), XP_002865782.1 (*Arabidopsis lyrata*), XP_006279783.1 (*Capsella rubella*), XP_004501828.1 (*Cicer arietinum*).

Basidiomycotas

EPQ53286.1 (*Gloeophyllum trabeum*), XP_007269166.1 (*Fomitiporia mediterranea*), XP_007366348.1 (*Dichomitus squalens*), EGN97430.1 (*Serpula lacrymans*), XP_007388213.1 (*Punctularia strigosozonata*), XP_007306061.1 (*Stereum hirsutum*), EIW77932.1 (*Coniophora puteana*), ESK95401.1 (*Moniliophthora roreri*), CCA67173.1 (*Piriformospora indica*), XP_007400380.1 (*Phanerochaete carnosa*), ETW76584.1 (*Heterobasidion irregulare*), XP_007366348.1 (*Dichomitus squalens*), XP_003031261.1 (*Schizophyllum commune*), EMD35912.1 (*Ceriporiopsis subvermispora*), XP_008044050.1 (*Trametes versicolor*), XP_001885889.1 (*Laccaria bicolor*), XP_001838292.2 (*Coprinopsis cinerea okayama*), XP_007332996.1 (*Agaricus bisporus*), XP_007353540.1 (*Auricularia delicata*), GAC76719.1 (*Pseudozyma antarctica*), CBQ71977.1 (*Sporisorium reilianum*), EJT46947.1 (*Trichosporon asahii var. asahii*), GAC96198.1 (*Pseudozyma hubeiensis*), EPQ27837.1 (*Pseudozyma flocculosa*), EST07712.1 (*Kalmanozyma brasiliensis*), ETS62978.1 (*Moesziomyces aphidis*).

Ascomycotas

XP_001222757.1 (*Mycothermus thermophilus*), XP_001259713.1 (*Neosartorya fischeri*), XP_001590406.1 (*Sclerotinia sclerotiorum*), XP_001804495.1 (*Phaeosphaeria nodorum*), XP_001931868.1 (*Pyrenophora tritici*), NP_596491.1 (*Schizosaccharomyces pombe*), XP_002174907.1 (*Schizosaccharomyces japonicus*), EEH04763.1 (*Ajellomyces capsulatus*), XP_003040990.1 (*Nectria haematococca*), EFQ29441.1 (*Colletotrichum graminicola*), XP_003176899.1 (*Nannizzia gypsea*), EFX02126.1 (*Grosmannia clavigera*), XP_003295416.1 (*Pyrenophora teres*), EHA27404.1 (*Aspergillus niger*), GAA86905.1 (*Aspergillus kawachii*), EHK22756.1 (*Trichoderma virens*), EHK42729.1 (*Trichoderma atroviride*), XP_003666337.1 (*Myceliophthora thermophila*), XP_003843847.1 (*Leptosphaeria maculans*), XP_003847820.1 (*Zymoseptoria tritici*), EJP64438.1 (*Beauveria bassiana*), EJT72939.1 (*Gaeumannomyces graminis* var. *tritici*), EKG13272.1 (*Macrophomina phaseolina*), ELQ38036.1 (*Pyricularia oryzae*), AGC83582.1 (*Aspergillus versicolor*), EMD68232.1 (*Bipolaris sorokiniana*), EMD93561.1 (*Bipolaris maydis*), EMF08215.1 (*Sphaerulina musiva*), EMR61726.1 (*Eutypa lata*), EMR84511.1 (*Botritis cinerea*), ENH77437.1 (*Colletotrichum orbiculare*), EOA91802.1 (*Setosphaeria turcica*), EON66846.1 (*Coniosporium apollinis*), EON95756.1 (*Phaeoacremonium minimum*), EPE05338.1 (*Ophiostoma piceae*), CCT69697.1 (*Fusarium fujikuroi*), EPS25670.1 (*Penicillium oxalicum*), EPX71580.1 (*Schizosaccharomyces octosporus*), ETI25985.1 (*Cladophialophora carrionii*), ETS84882.1 (*Pestalotiopsis fici*), XP_006673892.1 (*Cordyceps militaris*), XP_006691652.1 (*Thermochaetoides thermophila*), EUC31747.1 (*Bipolaris zeicola*), EUC44511.1 (*Bipolaris oryzae*), EUN23006.1 (*Bipolaris victoriae*), EWG55011.1 (*Fusarium verticillioides*), AAGE01071227.1 (*Cladophialophora yegresii*), EXJ74413.1 (*Cladophialophora psammophila*), EXJ86943.1 (*Capronia epimyces*), EXJ88152.1 (*Capronia coronata*), EXK27948.1 (*Fusarium oxysporum*), XP_007284185.1 (*Colletotrichum gloeosporioides*), EYE95713.1 (*Asperillus ruber*), XP_007600510.1 (*Colletotrichum fioriniae*).

Prokaryotes

YP_842090.1 (*Ralstonia eutropha*), YP_878325.1 (*Clostridium novyi*), YP_001681627.1 (*Heliobacterium modesticaldumIce*), NP_671412.1 (*Yersinia pestis*), YP_003843374.1 (*Clostridium cellulovorans*), YP_007085012.1 (*Oscillatoria acuminata*), WP_002579572.1 (*Clostridium butyricum*), WP_002596620.1 (*Clostridium colicanis*), WP_005213533.1 (*Clostridium celatum*), WP_007287091.1 (*Clostridium bartlettii*), WP_008472850.1 (*Lactobacillus gigeriorum*), WP_009448033.1 (*Parvimonas* sp.), WP_016206429.1 (*Clostridium sartagoforme*), WP_017302896.1 (*Spirulina subsalsa*), WP_017352896.1 (*Clostridium botulinum*), WP_017553431.1 (*Bacillus coagulans*), WP_021331467.1 (*Treponema socranskii*), WP_021802163.1 (*Clostridium intestinale*), WP_021875509.1 (*Clostridium chauvoei*), YP_008674399.1 (*Clostridium saccharobutylicum*), WP_022777093.1 (*Butyrivibrio* sp.), WP_026221520.1 (*Bacillus* sp.37MA), WP_011722321.1 (*Clostridium* sp), WP_006098082.1 (*Coleofasciculus chthonoplastes*), MEW5861306.1 (*Cyanobacteriota bacterium*), ACK69253.1 (*Gloeothece citriformis* PCC7424), WP_013323452.1 (*Gloeothece verrucosa*), AHW59668.1 (*Draconibacterium orientale*), OAB61127.1 (*Leptolyngbya valderiana*).
