# Supplementary material for: Mutational analysis of Phanerochaete chrysosporium´s purine transporter
Source: PLoS One. 2024 Oct 31;19(10):e0313174. doi: 10.1371/journal.pone.0313174 (PMC11527162; doi:10.1371/journal.pone.0313174)
Supplement: Attachment — Submitted filename: Response to Reviewers.docx [file pone.0313174.s010.docx]

**Dear Editor,**

**Regarding manuscript ID PONE-D-24-30056 - Mutational analysis of *Phanerochaete chrysosporium*´s purine transporter**, the authors would like to thank you for considering the publication of our work and present the revised version in which all comments have been taken into account.

**Academic editor.**

- *Reorganize the introduction section and concisely describe the main objective and contribution of the work.*
- *Avoid repeating the experimental approaches in the Results section and rather focus in describing the results obtained.*
- *Some caption figures need to be rewritten to summarize the information (Figure 1) and/or clarifying specific elements (Figs. 3 and 5).*
- *To improve the quality and resolution of Figure 1 and consider its organization and presentation.*

As suggested, the Introduction, Results and Materials & Methods sections were reorganised. The figures were also improved for the new version. Figure 1 (A and B) was separated into two independent figures (Figure 1 and Figure 2). Both new figures, as well as Figures 3 (Figure 4 in the new version) and 5 (Figure 6 in the new version), were redone and reorganised, improving quality and clarity. Each caption was revised and abbreviated.

- *Address the kinetical and structural concerns pointed, including the generation of 3D models of WT and mutants of PhZ employing AlphaFold.*

All of the kinetical concerns raised by the reviewer have been addressed. The statistical analysis has been performed as requested. The final results are conceptually consistent, with minor variations in the values resulting from differences between analysis methodologies. In the previous analysis, samples from each of the two trials performed were normalised to their respective wild-type values (3 replicates each) and then the results from both experiments were averaged. In the current analysis, all trials are analysed together (6 replicates). As a contribution of the new analysis, it was confirmed that the small differences in transport between some of the mutants and the wild-type PhZ strain were statistically significant for 8-azaguanine (as previously reported) but also for all other physiological substrates.

When we constructed the initial model in 2020, AlphaFold was still in its earlier stages and had not yet achieved the high accuracy it's known for today. As requested, we generated a new model using the latest version of AlphaFold and compared it with our initial homology model. The two models were similar, with an RMSD of 3.35 Å, but the AlphaFold model showed better stereochemical quality, particularly in the loop regions. For this reason, we decided not to present the results from the homology model and have used only the AlphaFold model for the revised docking calculations.

We appreciate the reviewers' comments, which, as outlined above, have been fully addressed. All sections, particularly the Introduction and Results (including figure captions), have been thoroughly corrected and reorganized in accordance with the reviewers' suggestions. We believe that these changes have significantly enhanced the quality of the manuscript and the presentation of the results. We are grateful for the constructive feedback and hope that the revisions meet the journal's standards and adequately respond to the reviewers' suggestions.

Sincerely,

**Mariana Barraco-Vega and Manuel Sanguinetti**

**Journal Requirements:**

*1. When submitting your revision, we need you to address these additional requirements. Please ensure that your manuscript meets PLOS ONE's style requirements, including those for file naming. The PLOS ONE style templates can be found at*

*https://journals.plos.org/plosone/s/file?id=wjVg/PLOSOne_formatting_sample_main_body.pdf and*

*https://journals.plos.org/plosone/s/file?id=ba62/PLOSOne_formatting_sample_title_authors_affiliations.pdf*

Done.

*2. Thank you for stating the following financial disclosure:*

Agencia Nacional de Investigación e Innovación under the codes PR_FCE_3_2013_1_100659 (research grant MBV) and POS_1097576 (doctoral fellowship MBV).

Comisión Académica de Posgrado CAP_2019 (doctoral finalisation fellowship MBV)

*Please state what* ***role the funders took*** *in the study.*

The funders had no role in study design, data collection and analysis, decision to publish, or preparation of the manuscript.

*3. Please upload a new copy of Figure 1****.*** Done.

*4. PLOS ONE now requires that authors provide the original uncropped and unadjusted images underlying all blot or gel results reported in a submission’s figures or Supporting Information files.*

The original image of the Western blot is provided as Supplementary Information (S1_raw_image).

***Reviewer #1: Comments to article PONE-D-24-30056***

***General remarks***

1. *The aim of the study should be made clear, as well as the reason why PhZ was chosen for mutational analysis, as this is very ambiguous in the introduction and leads to many questions that could affect the interpretation of the overall work.*

We have revised the introduction to clarify the aim of the study and explicitly stated the reasons for choosing PhZ for mutational analysis. This modification addresses the ambiguity and provides a clearer rationale for its selection.

2. *The description of the results should be carefully revised and rewritten. At several points in the manuscript, the description of the results seems more like a methodological description and in the figure captions the results are described.*

We have carefully revised and rewritten the description of the results to ensure they are clearly presented. We have separated methodological details from the results, and the figure captions now focus solely on describing the figures.

3. *In the results of the structural model, the description should focus more on the results obtained and not on comparisons with other models, as the notion of the real contribution of the work is lost. Comparisons with the results of other authors should be moved to the discussion.*

We have revised the results section to ensure that the contribution of our work is clearly highlighted. While the comparison of our results with those from other studies is discussed in more depth in the discussion section, we believe it is necessary to include some mentions in the results section to validate the model obtained.

4. *Express decimal amounts with periods and not commas, for example, 0.8 instead of 0,8 for clarity.*

Done.

***Specific comments***

*Line 34. Replace "purines" with "purine.* Done.

*Line 74. Has the structure of the AzgA protein of Aspergillus nidulans or any other microorganism been previously crystallized?*

A sentence was added in this regard.

*Lines 91-104. The objective of the study is not well defined, so the following questions are raised:*

*1. Why was the mutational analysis not performed directly on the AzgA protein of Aspergillus nidulans? Is its structure and the residues involved in purine transport already known? If so, this should be indicated in the introduction.*

*2. Why was PhZ chosen among the 11 AzgA-type proteins? What is the importance of analyzing this protein? If the goal is to understand the structural differences between UapA/C and AzgA proteins that lead to substrate differences, as inferred in lines 82-84, why not perform a mutational analysis of UapA/C residues involved in purine transport, or of differential residues with AzgA, if the crystal structure of UapA/C is known*

Answers 1 and 2. These were addressed in the new version of the manuscript. Responding to the reviewer, we would like to say that although part of our group was responsible for the identification of the *A. nidulans* AzgA protein and its first characterisation, our research is currently focused on basidiomycetes fungi. In this study, then, we chose to work with PhZ to extend studies of the AzgA-like subfamily to Basidiomycetes since most studies focus on Ascomycetes. Additionally, the requested information regarding the structure of AzgA and the residues involved in purine transport has been incorporated into the introduction of the revised manuscript. While one of the objectives is to understand the structural differences between UapA/C and AzgA proteins that lead to differences in substrate specificity, in this work, we did not focus on analysing the UapA/C residues involved in purine transport, as this approach was already addressed in Krypotou et al. 2014. The aim of this study is to contribute to the characterization of the AzgA-like family from a different perspective. Further clarification on the importance of analysing PhZ has been incorporated into the new version of the manuscript.

*Lines 91-114. I recommend rewriting this paragraph to give fewer details of the results and to concisely describe the contribution of the work.* Done.

*Lines 111-112. The hypothesis is very ambiguous.*

We hope to have better defined the hypothesis***.***

*Table S1. Correct the description of the strain phZ_V58A*.* Done.

*Line 126. Put the formula for ammonium tartrate in parentheses, not just ammonium.* Done.

*Line 127. Indicate which purines and where they were acquired from.* Done.

*Line 128. Indicate why these concentrations of 8-azaguanine and oxypurinol were used to assess toxicity. If a reference exists as background, include it. If not, provide adequate justification.*

Done. We added the reference.

*Line 166. Recommended by...?* Added.

*Line 257. The representation of the conserved motifs in a table in Figure S2 is very confusing and difficult to understand. I suggest generating a figure with the alignment of the sequences.*

Figure S2 has been reorganised for easier reading and understanding. A file was generated with the identification of the sequences used (S1 Appendix).

*Line 264. The figure caption in Figure 1 is too long. It should be more concise and describe the figure in the text in more detail instead of too much description in the figure caption. I suggest splitting Figure 1 into 2 parts so that Figure 1 B is Figure 2 so that it can be analyzed separately as it discusses the mutants generated.* Done.

*Line 303-311. Part of the information in this section corresponds to the methodology.* Done.

*Line 334. I consider that figure 2 should be better described in the text, not only in the figure caption, since the figure caption indicates why only the photos of the hx results are included and not those of adenine and guanine, but this is not mentioned in the text, or I suggest changing figure 2 for figure S3 but including the control in which AzgA is expressed. Photographs of the growth of the WT strain of Aspergillus nidulans under the different conditions should also be included in this figure.*

A sentence describing both, what is shown in each Figure 2 (Fig 3 in the new version) and S3 Fig, and the criteria used, was added to the text. The description of the results observed in both figures was also completed in the text.

*Line 372. Fig.3. Indicate what %V means.* Done.

*Line 400. Indicate with which substrate differences were observed.*

This part has been reorganised to clarify the description.

***Reviewer #2:***

*The authors present a complete characterization of several single mutants of purine transporter from Phanerochaete chrysporium. The manuscript will be of interest to those involved in the study of purine transporters (substrate specificity and transport efficiency) and in general in protein structure and function analysis. The work was performed applying cutting-edge techniques that supports the findings and conclusions stated. However, there are minor details that deserve attention.*

*1.- The IC50 value depends on substrate concentration used, therefore, how the concentration of radiolabeled substrate used (at least 10 times lower than the Km value) affected the Ki determination?*

As recommended in Krypotou et al. 2014, the K_m/i_ is calculated using the formula K_i_ = IC50 / (1 + [S]/K_m_), where [S] is the concentration of the radiolabelled substrate. In our assays, K_i_ values equal IC50 values since the [S] is very low (at least 10-fold lower than the K_m_ value). The method is in fact identical to the one for K_m_ determination, but stock solutions are prepared using a mixture of fixed radiolabelled substrate and increasing concentrations of non-radiolabelled substrates.

*2.- In the same context, please explain in the text the meaning of Km/i and include a table as supplementary material with the values of IC50 obtained and indicate the value of Km used.*

In the revised manuscript, we have included an explanation of the meaning of K_m/i_ within the text. The values of K_m/i_, which under our experimental conditions are equivalent to IC50, are presented in Figure 5. Therefore, we have not included an additional supplementary table, as these data are already displayed in the figure.

*3.- Why do not try to use an AI software such as Alphafold to generate the 3D model of the transporter?*

*4.- Please include the stereochemical evaluation of the final model used for structural and docking studies.*

Answer 3 and 4. When we constructed the initial model in 2020, AlphaFold was still in its earlier stages and had not yet achieved the high accuracy it's known for today. Back then, homology modelling was a widely accepted and reliable approach for generating 3D models, especially for complex systems like transporters. As requested, we generated a new model using the latest version of the software and compared it with our initial model. Upon comparison, we found that the RMSD (Root Mean Square Deviation) between the AlphaFold model and the original is 3.35 Å, with the largest differences mainly located in the loop regions, suggesting that our first model was quite close to the actual structure.

Finally, as requested, we performed a stereochemical comparison of both models using PROCHECK. Most residues fall within favourable regions of the Ramachandran plot, and the G-factors are close to zero, indicating good overall stereochemical quality. The AlphaFold model has slightly better values than our initial one (see S5 Fig in the manuscript and table below).

Taking it all together, we decided it is unnecessary to include both models in the manuscript, and therefore, we will only present the AlphaFold model, redoing the docking calculations accordingly.

*5.- If the protein is a transporter, why did not perform the MD simulation using a membrane instead of an aqueous system?*

The molecular dynamics (MD) simulation was performed with the primary goal of refining the model and improving its structural quality, rather than studying the protein’s mechanism or transport function. For model refinement purposes, a simpler aqueous system was sufficient. Using a membrane environment would be more appropriate if the objective were to investigate the protein's interactions with the lipid bilayer, its transport mechanism, or other functional aspects. In this case, the focus was solely on optimizing the structural features of the model

*6.- In would be desirable to generate the corresponding 3D models at least from the most interesting mutants to perform docking studies to explain the experimental data and not only with the native protein structure.*

Done. We generated AlphaFold model and performed docking studies for the L124 mutant, as this residue is part of the binding site and exhibited significant differences in transport activity. In contrast, I388, which was also considered, did not show any differences in transport and was therefore not included in the docking analysis.

***Reviewer #3:***

*In this work, the authors describe a structure-function study of the purine transporter PhZ from Phanerochaete chrysosporium, initially focused on 4 conserved motifs of the NAT family, that unveiled a set of amino acid residues critical for transport activity and substrate specificity.*

***General comments:***

*This work utilizes a PhZ 3D model constructed by homology modeling using the crystal structure of UapA, further refined by molecular dynamics. Given the recent advances in 3D structure prediction by AlphaFold it would be interesting to include some data on this model, and compare it with the model used in this work. If the authors have already tested it, they could include some information in the manuscript. If not, they could retrieve the predicted structure by AlphaFold2.*

As requested, we generated a new model using the latest version of the AlphaFold software and compared it with our initial model. Upon comparison, we found that the RMSD (Root Mean Square Deviation) between the AlphaFold model and the original is 3.35 Å, with the largest differences mainly located in the loop regions, suggesting that our first model was quite close to the actual structure.

Finally, as requested, we performed a stereochemical comparison of both models using PROCHECK. Most residues fall within favourable regions of the Ramachandran plot, and the G-factors are close to zero, indicating good overall stereochemical quality. The AlphaFold model has slightly better values than our initial one (see S5 Fig in the manuscript and table below).

Taking it all together, we decided it is unnecessary to include both models in the manuscript, and therefore, we will only present the AlphaFold model, redoing the docking calculations accordingly.

*It is important that the authors include* ***statistical analysis*** *of the data to determine if, in fact, there are* ***significant differences*** *in some of the presented results, as sometimes stated in the manuscript.*

We have performed a statistical analysis to determine whether there are significant differences in the results, as stated in the manuscript. The analysis included one-way ANOVA, followed by Tukey's post-hoc test, to compare group means. For non-parametric data, we applied the Kruskal-Wallis test followed by Dunn's post-hoc test with Bonferroni and Holmes correction. The procedure is detailed in the Materials and Methods section of the revised manuscript.

**Abstract**

*The authors should state here why the A418 residue is pivotal and its location in the protein.* Done.

**Introduction**

*Line 48: Please clarify if AzgA subfamily lacks homologs in animals or structural homologs.*

Corrected. There are no homologues of the AzgA protein in animals. The word "structural" was removed.

*Line 84: Papakostas and colleagues (2013), the authors should include the reference number.*

Done. The reference was moved from the end to the beginning of the paragraph, after the cited authors.

*Line 87-90: please include here what were the main findings of this study, as they are important for the work presented here*.

Done. A paragraph was added at the end of the Introduction to this effect.

*Line 108: The reference regarding the post-translational regulation of the PhZ transporter is missing.* Done.

**Materials and Methods**

*Line 177: Why weren’t the Km values calculated directly with the labeled substrate at different concentrations? Why did the authors prefer to use a fixed concentration of labelled hypoxanthine and various concentrations of unlabelled hypoxanthine to determine the Ki?*

We chose to determine K_i_ primarily because of the cost of labelled substrates. K_i_ allows several substrates to be evaluated by comparative analysis within the same system using a single labelled substrate, as described by Krypotou and Diallinas (Fungal Genet. Biol. 2014, 63, 1–8).

*Line 186: What was the specific activity of the labelled hypoxanthine?* Added.

**Results**

*Line 246: The authors should include the accession of all the sequences used in the alignment in the supplementary data.*

The requested file has been added (S1 Appendix)

*Lines 259-262: where are located motifs 1 and 3? This will help the reader understand why this study discarded these motifs.* Done.

*Figure 1 B) This image could be improved as several mutations overlap. Instead of showing the complete alignment, repeating the previous image, the authors could use a simplified scheme where the position of each amino acid residue is reported regarding the PhZ protein, evidencing the effect of each mutation.*

Done. The figure was simplified as suggested by the reviewer.

*Line 305: Please clarify here the name of the strain and the main purine transporters deleted.* Done.

*Line 305: The authors state that the GFP did not affect the transporter activity, as the same growth phenotypes were observed in previous work. However, did the authors test the kinetic parameters of hypoxantine transport without the C- terminus GFP fusion? This method is more accurate to determine if the GFP tag affects protein activity.*

Although we did not determine the K_m_ constant for the version of PhZ without GFP, we compared the initial uptake rates and found no significant differences between PhZ-GFP and the version without GFP. These results have been included in Figure S2 and clarified in the text of the revised manuscript. While we acknowledge that the approach used may not be the most precise, considering that the study only compares strains with GFP, we believe this approach is sufficient for the purposes of this work.

*Line 319: Instead of “ less than that of the wild-type” consider “ is less than that of cells expressing the wild-type”* Done.

*line 330: include the growth phenotypes of this strain as mentioned for the previous strains.* Done.

*line 360: modify the efficiency is not very clear, as it can be an increase or decrease in transport activity. In this case the phenotype suggests an increase in transport capacity.* Done.

*line 361: please state the hypoxanthine concentration used to determine the initial uptake rate, and why this concentration was chosen.* Done.

*line 368: Statistical analysis should be applied to this data to evaluate which are significantly different.*

We have performed a statistical analysis to determine whether there are significant differences in the results, as stated in the manuscript. The analysis included one-way ANOVA, followed by Tukey's post-hoc test, to compare group means. For non-parametric data, we applied the Kruskal-Wallis test followed by Dunn's post-hoc test with Bonferroni and Holmes correction. The procedure is detailed in the Materials and Methods section of the revised manuscript.

*line 385 (figure 4): no SD are visible in the data concerning the uptakes of the L214M mutant. Is this correct?* Corrected.

*line 389: The authors state that this mutant has lower transport capacity, but they only report the assay where they tested one concentration. The Vmax of the transporter was not determined to evaluate transporter capacity.*

We agree that without determining V_max_, it is not accurate to refer to transport capacity. When we previously mentioned transport capacity, we were referring to transport efficiency. We have reviewed all instances where the term 'capacity' was used and have corrected them in the revised version of the manuscript.

*lines 394-397: This depends on the concentration of the unlabelled substrate. What were the conditions tested?*

As recommended in Krypotou et al. 2014, uptake assays were performed using a mixture with a fixed concentration of labelled hypoxanthine (0.2 µM) and an excess amount of unlabelled substrate (1 mM). This has now been correctly indicated in the Materials and Methods section of the revised manuscript.

*Line 415 (Fig 5) I consider it not easy to visualize the degree of inhibition of each substrate for the different mutants. I believe it would be easier if the data were presented differently, first for the WT and then for each mutant as follows: first, the V of hypoxanthine uptake w/o any inhibitor (100%), then with unlabelled hypoxanthine, and then the corresponding V % for all the tested inhibitors.*

*We have revised Figure 5 (now Figure 6 in the new version), incorporating all the suggested modifications. Additionally, to improve visualization, we have included only the results for the physiological substrates.*

*Also, statistical analysis should be presented here.* Done.

**Discussion:**

*Line 616: Please explain how an increase in the intrinsic stability of the mutant protein would decrease the hypoxanthine uptake rate.*

The explanation was added.
